# Drug Repositioning for the Prevention and Treatment of Chemotherapy-Induced Peripheral Neuropathy: A Mechanism- and Screening-Based Strategy

**DOI:** 10.3389/fphar.2020.607780

**Published:** 2021-01-14

**Authors:** Shota Yamamoto, Nobuaki Egashira

**Affiliations:** ^1^ Department of Lipid Signaling, National Center for Global Health and Medicine, Tokyo, Japan; ^2^ Department of Pharmacy, Kyushu University Hospital, Fukuoka, Japan

**Keywords:** chemotherapy, chemotherapy-induced peripheral neuropathy, drug repositioning, neuropathic pain, oxaliplatin, paclitaxel

## Abstract

Chemotherapy-induced peripheral neuropathy (CIPN) is a severe adverse effect observed in most patients treated with neurotoxic anti-cancer drugs. Currently, there are no therapeutic options available for the prevention of CIPN. Furthermore, few drugs are recommended for the treatment of existing neuropathies because the mechanisms of CIPN remain unclear. Each chemotherapeutic drug induces neuropathy by distinct mechanisms, and thus we need to understand the characteristics of CIPN specific to individual drugs. Here, we review the known pathogenic mechanisms of oxaliplatin- and paclitaxel-induced CIPN, highlighting recent findings. Cancer chemotherapy is performed in a planned manner; therefore, preventive strategies can be planned for CIPN. Drug repositioning studies, which identify the unexpected actions of already approved drugs, have increased in recent years. We have also focused on drug repositioning studies, especially for prevention, because they should be rapidly translated to patients suffering from CIPN.

## Introduction

Chemotherapy-induced peripheral neuropathy (CIPN) is a severe and dose-limiting adverse effect of neurotoxic chemotherapeutic agents. CIPN can limit the dosages and choices of drugs, and in serious cases, it leads to discontinuation of treatment. Anti-cancer drugs that often cause CIPN include platinum derivatives, taxanes, vinca alkaloids, and bortezomib. Most cases of CIPN are characterized by numbness, tingling, pain, and impaired sensory functions in the hands and feet. In particular, pain symptoms indicate mechanical and cold allodynia, and the time of onset can range from acute (within hours) to chronic (after repeated treatments) (Mantyh, 2006; Park et al., 2013). According to the American Society of Clinical Oncology (ASCO) clinical practice guidelines released in 2014 and 2020, there are no highly recommended agents for the prevention or treatment of existing CIPN (Hershman et al., 2014; Loprinzi et al., 2020).

Numerous studies using rodent models have reported various events occurring in the dorsal root ganglia (DRG) and the spinal cord after chemotherapy, such as ion channel dysfunction, axonal degeneration of the sciatic nerve, impaired mitochondrial function, and excessive oxidative stress. Of note, taxanes cause more severe inflammatory events compared with platinum derivatives. Despite similar phenotypes, each chemotherapeutic drug induces CIPN via different mechanisms. Therefore, it is important to understand the drug-specific pathogenic mechanisms and to develop measures against each CIPN.

In clinical practice, several drugs are used for CIPN, such as pregabalin, tricyclic antidepressants, and serotonin-noradrenaline reuptake inhibitors. In addition, there are now many clinical trials investigating the effectiveness of approved drugs against CIPN (Brewer et al., 2016; Cavaletti and Marmiroli, 2020). However, most of these available drugs were shown to have poor efficacy, except for duloxetine, which is moderately recommended for the treatment of existing CIPN (Hershman et al., 2014; Loprinzi et al., 2020). Although several drugs, such as metformin and venlafaxine were reported to be effective against CIPN, confirmation is needed (Durand et al., 2012; El-Fatatry et al., 2018). Therefore, it is important to elucidate novel mechanisms based on target molecules for drug development. However, safety concerns can prevent validation of the analgesic potential of candidate agents against CIPN as well as other pain symptoms. Drug repositioning represents an alternative approach to mitigate for safety issues (Sisignano et al., 2016b). Drug repositioning studies attempt to identify unexpected actions with approved drugs used in clinical practice. This approach can reduce the duration and cost of drug development because the pharmacokinetics and safety of approved drugs have already been examined in humans. Therefore, drug repositioning is rapidly translated to patients who are suffering from CIPN.

Neuropathic pain is often caused by damage to the nervous system such as trauma, cancer, diabetes, virus infection, autoimmune disease, or chemotherapy (Colloca et al., 2017). Except for chemotherapy, most of these conditions occur unexpectedly, and therefore a number of studies are investigating the mechanisms of intractable pain to develop novel therapeutic drugs. However, because cancer chemotherapy is scheduled and administered via a regimen, CIPN is the only neuropathic pain that can be prevented. Therefore, drug-repositioning studies are a promising strategy to manage preventable neuropathic pain.

In this review, we summarize the growing evidence surrounding oxaliplatin- and paclitaxel-induced CIPN, including the mechanisms of onset and potential treatments in rodent models. We also highlight recent findings, with a focus on drug repositioning studies. Detailed information about the developmental mechanisms or clinical aspects of CIPN has been reported in previous excellent reviews (Cavaletti and Marmiroli, 2010; Hershman et al., 2014; Sisignano et al., 2014; Loprinzi et al., 2020).

## Methodology

We searched using the terms “oxaliplatin neuropathy,” “oxaliplatin neurotoxicity,” “paclitaxel neuropathy,” and “paclitaxel neurotoxicity” in PubMed. Articles related to CIPN pathology and drug repositioning studies for the prevention and/or treatment of CIPN using cellular models or animal models were manually selected based on originality and relevance to the scope of this review. We excluded the articles written in a language other than English.

## Mechanisms of CIPN

### Oxaliplatin-Induced CIPN

**FIGURE 1:**
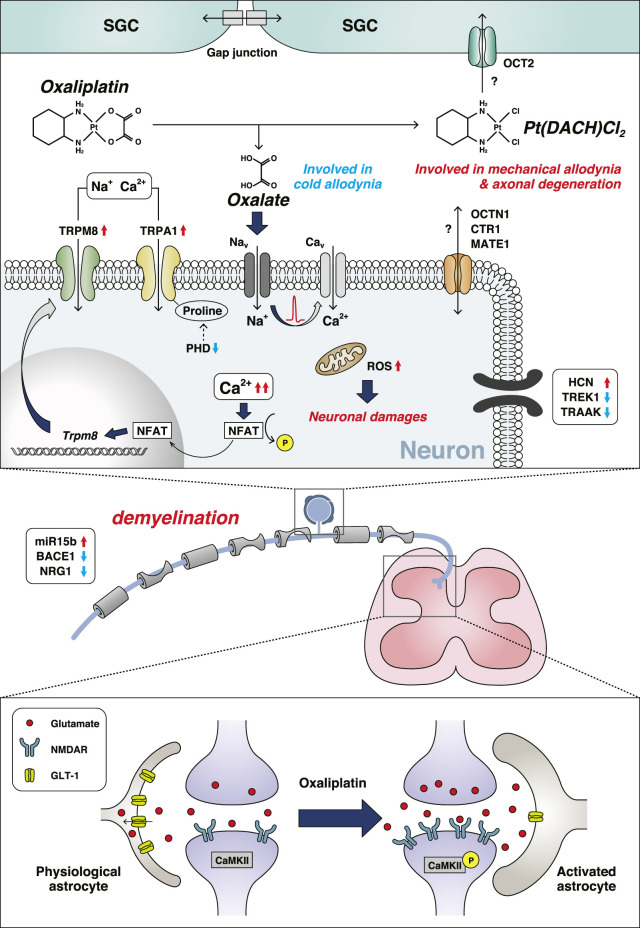
Mechanisms of oxaliplatin-induced peripheral neuropathy. Oxaliplatin is metabolized to oxalate and Pt (DACH)Cl_2_, under the presence of physiological chloride. The red up arrows indicate an increase and/or activation, and the blue down arrows indicate a decrease and/or inhibition. Abbreviations: β-secretase 1 (BACE1); Ca^2+^/calmodulin dependent protein kinase II (CaMKII); voltage-gated calcium channel (Cav); copper transporter 1 (CTR1); glutamate transporter 1 (GLT-1); hyperpolarization-activated cyclic nucleotide-gated cation channel (HCN); multidrug and toxic compound extrusion 1 (MATE1); voltage-gated sodium channel (Nav); nuclear factor of activated T-cell (NFAT); N-methyl-d-aspartate receptor (NMDAR); neuregulin 1 (NRG1); organic cation transporter 2 (OCT2); organic cation/carnitine transporter 1 (OCTN1); prolyl hydroxylase (PHD); reactive oxygen species (ROS); satellite glial cell (SGC); potassium channel subfamily K member 4 (TRAAK); potassium channel subfamily K member 2 (TREK1); transient receptor potential channel (TRP).

Oxaliplatin is a platinum-based anticancer drug widely used as a first-line treatment for colorectal, gastric, and pancreatic cancers. Although oxaliplatin is highly effective for the treatment of cancers, it often causes severe neuropathic symptoms that can be divided into two types: acute-onset cold hypersensitivity within a few days after treatment and chronic sensory neuropathy including tactile allodynia and numbness occurring after repeated treatments. Oxaliplatin is metabolized to oxalate and dichloro (1,2-diaminocyclohexane)platinum (II) (Pt (DACH)Cl_2_), which are involved in acute cold hypersensitivity and chronic neuropathy, respectively (Sakurai et al., 2009). Oxaliplatin affects the expression level and/or function of ion channels and induces excessive neuronal excitation, oxidative stress, and neurodegeneration through multiple mechanisms (Figure 1).

#### Ion Channel Dysregulation

Oxaliplatin-induced cold hypersensitivity is mainly caused by the alteration of ion channel activity, and is often recognized as a channelopathy. Oxalate is a chelator of intracellular Ca^2+^ (Grolleau et al., 2001) that disturbs neuronal membrane potentials followed by the dysregulation of voltage-gated ion channel activity. Oxaliplatin treatment alters the activities of voltage-gated ion channels and ligand-gated ion channels such as transient receptor potential (TRP) channels. Cold responsible subtypes, TRP melastatin 8 (TRPM8) and TRP ankyrin 1 (TRPA1), also contribute to oxaliplatin-induced cold hypersensitivity (Gauchan et al., 2009; Nassini et al., 2011; Zhao et al., 2012; Trevisan et al., 2013). TRPM8 expression in the DRG is upregulated early after oxaliplatin (or oxalate) infusion via a nuclear factor of activated T-cell (NFAT)-dependent mechanism, which is attenuated by blocking L-type calcium channels (Kawashiri et al., 2012). Furthermore, a recent study demonstrated that oxaliplatin inhibited prolyl hydroxylase, an enzyme that hydroxylates proline in the N-terminal ankyrin repeat of TRPA1, which leads to the enhanced sensitivity of TRPA1 (Miyake et al., 2016). In addition, it has been reported that oxaliplatin treatment lowers the cytosolic pH of DRG neurons through binding to neuronal hemoglobin, which acts as a proton buffer. This acidification sensitizes TRPA1, which in turn leads to the development of cold hypersensitivity after oxaliplatin administration (Riva et al., 2018; Potenzieri et al., 2020).

Several other cold-sensing ion channels are also involved in oxaliplatin-induced cold neuropathy (Pereira et al., 2014; Resta et al., 2018). The expression of two distinct potassium channels, TREK1 and TRAAK, are lowered by oxaliplatin. In contrast, oxaliplatin increases the expression of hyperpolarization-activated channels (also known as HCN channels) (Descoeur et al., 2011). This remodeling of ion channel expression patterns promotes the development of oxaliplatin-induced cold hypersensitivity.

#### Mitochondrial Dysfunction and Oxidative Stress

Oxaliplatin causes morphological changes and functional deficits of mitochondria. The number of swollen and vacuolated mitochondria was increased in the peripheral nerves after oxaliplatin treatment (Xiao et al., 2012). In addition, multiple oxaliplatin administrations decrease O_2_ consumption and ATP production in the sciatic nerve, which is associated with the reduced activity of the mitochondrial respiratory chain complex I/II (Zheng et al., 2011). Acetyl-l-carnitine, an antioxidant with protective effects on mitochondria, ameliorated oxaliplatin-induced CIPN in rats. Oxaliplatin also caused the loss of mitochondrial membrane potential in Schwann cells (Imai et al., 2017).

Oxaliplatin treatment was reported to activate nuclear factor-erythroid-2-related factor 2 (Nrf2) signaling, which plays a crucial role in mitochondrial function, and Nrf2 knockout mice developed aggravated neuropathic symptoms after oxaliplatin administration (Yang et al., 2018).

#### Neuronal Damage

Platinum accumulation was correlated with neuronal damage induced by oxaliplatin treatment (Holmes et al., 1998; Cavaletti et al., 2001). Several transporters were reported to be involved in platinum transport in the DRG. Among them, the overexpression of organic cation transporter 2 (OCT2) markedly increased the cellular uptake of oxaliplatin and platinum-DNA adduct formation. Importantly, genetic knockout or pharmacological inhibition of OCT2 protected mice from developing acute neuropathic symptoms after oxaliplatin administration (Sprowl et al., 2013). Surprisingly, a recent study reported that OCT2 was mainly expressed on satellite glial cells (SGCs), which suggests a critical interaction between sensory neurons and peripheral glial cells (Huang et al., 2020). However, the cell type responsible for causing oxaliplatin-mediated neuronal damage should be verified by the cell type-specific regulation of OCT2 expression with genetic modification tools. In addition, a recent study showed that organic cation/carnitine transporter 1 and multidrug and toxic compound extrusion 1 also play roles in the influx/efflux of oxaliplatin in the DRG, respectively (Jong et al., 2011; Nishida et al., 2018; Fujita et al., 2019).

Oxaliplatin uptake in the DRG results in neuronal damage, such as excessive oxidative stress followed by axonal degeneration in the sciatic nerve of rodent models. Hypomyelination was also observed in the sciatic nerve, and this reduction in myelin sheath integrity might be related to alterations in the miR-15b, β-secretase 1, and cleaved neuregulin 1 pathways (Tsutsumi et al., 2014; Ito et al., 2017).

#### Alteration of Central Nervous System Functions

Most chemotherapeutic drugs including oxaliplatin have limited permeability across the blood-brain barrier; however, oxaliplatin can induce central nervous system (CNS) dysfunction. Oxaliplatin increases the expression of *N*-methyl-d-aspartate receptor (NMDAR) followed by the phosphorylation of Ca^2+^/calmodulin dependent protein kinase II in the spinal cord, which might contribute to the development of oxaliplatin-induced CIPN (Mihara et al., 2011; Shirahama et al., 2012). Moreover, an increase in extracellular glutamate concentration and a decrease in glutamate transporter 1 expression were observed in the spinal cords of oxaliplatin-treated rats and were involved in the development of mechanical allodynia (Chelini et al., 2017; Yamamoto et al., 2017).

Oxaliplatin-induced CIPN might also involve astrocytic activation in the spinal cord. Activated astrocytes increase the formation of gap junctions, which might result in pain hypersensitivity (Di Cesare Mannelli et al., 2013a; Yoon et al., 2013). However, oxaliplatin-induced glial cell activation might not be severe.

### Paclitaxel-Induced CIPN

**FIGURE 2:**
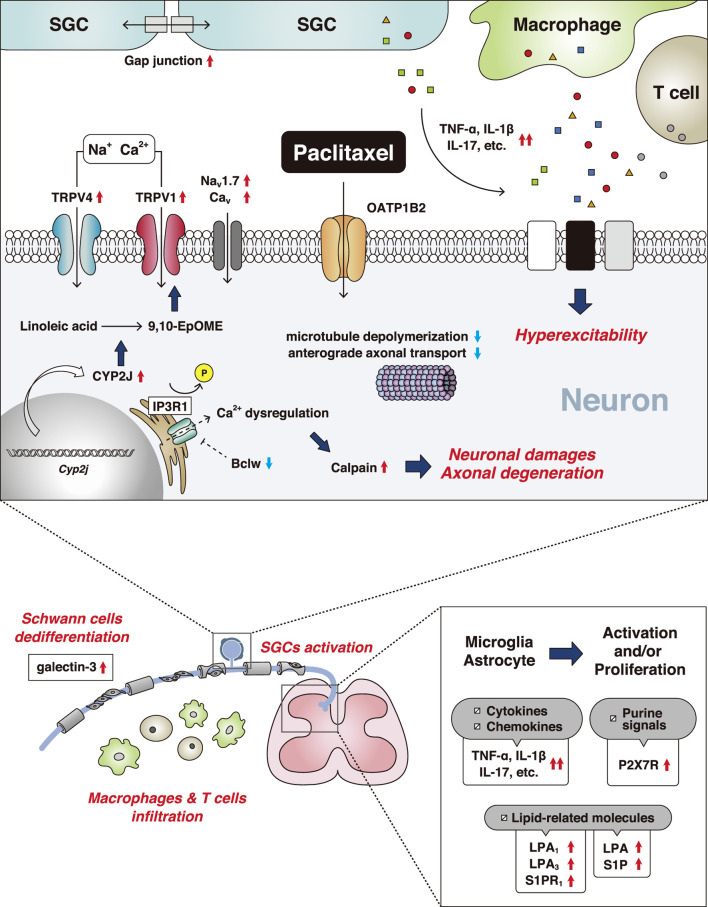
Mechanisms of paclitaxel-induced peripheral neuropathy. The red up arrows indicate an increase and/or activation and the blue down arrows indicate a decrease and/or inhibition. Abbreviations: 9,10-epoxy-12Z-octadecenoic acid (9,10-EpOME); bcl-2-like protein 2 (Bclw); voltage-gated calcium channel (Cav); cytochrome-P450-epoxygenase 2J (CYP2J); interleukin (IL); inositol 1,4,5-trisphosphate receptor 1 (IP3R1); lysophosphatidic acid (LPA); voltage-gated sodium channel (Nav); organic anion-transporting polypeptide B2 (OATP1B2); P2X purinoceptor 7 (P2X7R); sphingosine 1-phosphate (S1P); satellite glial cell (SGC); tumor necrosis factor (TNF); transient receptor potential channel (TRP).

Paclitaxel is an antineoplastic drug extracted from *Taxus brevifolia* and is used to treat several malignancies, including breast cancer, non-small cell lung carcinoma, and stomach cancer. Paclitaxel has a microtubule-targeting effect, which interferes with the construction of cell structures and function of microtubules. Microtubules have important roles in neuronal function, and thus paclitaxel often induces peripheral neuropathy. Paclitaxel administration causes ion channel dysfunction, axonal degeneration, and inflammatory events (Figure 2).

#### Neuronal Degeneration and Dysfunction of Axonal Transport

A recent study identified the murine solute carrier organic anion-transporting polypeptide 1B2 (OATP1B2) as a transporter that uptakes paclitaxel into DRG neurons (Leblanc et al., 2018). Inhibition of OATP1B2 prevented paclitaxel-induced sensory hypersensitivity. In paclitaxel-treated animals, the upregulation of ATF3, a marker of nerve injury, was observed in DRG neurons (Peters et al., 2007; LoCoco et al., 2017). Moreover, paclitaxel induced axonal degeneration in the sciatic nerve (Cavaletti et al., 1995). Wallerian Degeneration Slow mice are resistant to neuronal dysfunction after paclitaxel injection, indicating neurodegeneration is required for the development of paclitaxel-induced CIPN (Wang et al., 2002).

Cumulative evidence indicates that calcium-dependent calpain proteases are critical regulators leading to axonal degeneration (Wang et al., 2004; Boehmerle et al., 2007). Paclitaxel dephosphorylates axonal inositol 1,4,5-trisphosphate receptor 1 (IP3R1), which induces Ca^2+^ dysregulation, resulting in the activation of calpain proteases and the initiation of axonal degeneration (Pease-Raissi et al., 2017). In addition, paclitaxel interfered with anterograde kinesin-based axonal transport but not dynein-based retrograde transport (Bobylev et al., 2015; Smith et al., 2016). IP3R1 is inhibited by Bclw (a Bcl2 family member); however, paclitaxel impaired the axonal transport of Bclw mRNA, which triggered the axonal degeneration cascade (Pease-Raissi et al., 2017).

#### Inflammation and Non-neuronal Cell Activation

Paclitaxel causes multiple inflammatory events compared with oxaliplatin, and a variety of non-neuronal cells contribute to its pathogenesis. In the peripheral nervous system, macrophages infiltrate into the DRG and release several inflammatory cytokines, such as tumor necrosis factor-α and interleukin-1β (IL-1β) (Ledeboer et al., 2007; Zhang et al., 2016). Macrophages and SGCs are activated by paclitaxel (Peters et al., 2007). Gap junction-mediated coupling between SGCs was increased after paclitaxel treatment and the activation of SGCs affected neuronal activity mediated by IL-17 and/or other cytokines and glial transmitters (Warwick and Hanani, 2013; Luo et al., 2019a). Recently, the dysregulation of Schwann cells was shown in paclitaxel-induced CIPN (Wozniak et al., 2018). Paclitaxel treatment impaired Schwann cells by their dedifferentiation into an immature state, which might disrupt communication between neurons and Schwann cells (Imai et al., 2017). Furthermore, the number of CD8^+^ T cells was increased in the DRG of paclitaxel-treated mice, contributing to recovery from neuropathic symptoms via IL-10-mediating signaling (Krukowski et al., 2016).

In the CNS, microglial and astrocytic activation occur in the spinal dorsal horn (Peters et al., 2007; Zhang et al., 2012; Luo et al., 2019a). Similar to the DRG, several inflammatory cytokines and chemokines were increased, which modulated dorsal horn neuronal activities (Li et al., 2015; Manjavachi et al., 2019). The increased expression of microglial purinoceptor P2X7 receptor enhanced CCL3-CCR5 signals involved in mechanical allodynia (Ochi-ishi et al., 2014). Several species of lipid mediator were also increased in the spinal cord in paclitaxel-induced CIPN. Lysophosphatidic acid (LPA) and sphingosine 1-phosphate (S1P) were required for the development of paclitaxel-induced neuropathic pain via the receptors LPA_1_/LPA_3_ and S1PR_1_, respectively (Janes et al., 2014; Uchida et al., 2014).

#### Ion Channel Dysregulation

In contrast to oxaliplatin, paclitaxel indirectly alters ion channel activity by endogenous mediators and their signaling pathways, which leads to neuropathic pain. Several voltage-gated ion channels such as T-type Ca^2+^ channel and Na_v_1.7 are involved in paclitaxel-induced CIPN. Furthermore, the inhibition of these channels attenuated mechanical allodynia (Flatters and Bennett, 2004; Li et al., 2018).

TRP channels also have important roles in paclitaxel-induced CIPN (Alessandri-Haber et al., 2004; Chen et al., 2011; Hara et al., 2013; Boehmerle et al., 2018). Agonism of kinin receptors sensitized TRP vanilloid 4 (TRPV4) via a PKC-dependent pathway, which is involved in paclitaxel-induced peripheral neuropathy (Costa et al., 2018). Paclitaxel increased several lipid metabolites in the DRG, especially 9,10-EpOME, a metabolite of linoleic acid, which was upregulated via the elevation of cytochrome-P_450_-epoxygenase CYP2J. Sensitization of TRPV1 channels in DRG neurons by this lipid mediator contributed to the development and maintenance of paclitaxel-induced pain hypersensitivity (Sisignano et al., 2016a). Furthermore, deficiency in another TRP channel family member, TRPM2, had a protective effect against paclitaxel-induced CIPN (So et al., 2015).

## Drug Repositioning Study for the PREVENTION AND TREATMENT OF CIPN

Drug development is costly and lengthy. Many novel analgesics are dropped during clinical trials because of their lack of efficacy or safety problems (Sisignano et al., 2016b). Drug repositioning has been proposed for the discovery of new uses for approved drugs; therefore, it might be an alternative strategy to the high cost and time required for drug development whilst maintaining safety. Furthermore, drug repositioning is an efficient strategy for the treatment of drug-induced adverse effects, such as CIPN.

Recently, increasing numbers of studies have reported the preventive effects of approved drugs for CIPN (Table 1). These drug repositioning studies can be divided into “mechanism-based” (known developmental mechanism of CIPN and the action mechanism of approved drugs) or “screening-based” studies (utilizing chemical libraries including approved drugs to discover the novel action of a drug for the prevention and/or treatment of CIPN).

**TABLE 1:** Potential candidates of drug repositioning for CIPN identified in preclinical studies Cytochrome-P_450_-epoxygenase 2J (CYP2J), High-mobility group box 1 (HMGB1), *N*-methyl-d-aspartate receptor (NMDAR), nuclear factor-erythroid-2-related factor 2 (Nrf2), organic anion-transporting polypeptide B2 (OATP1B2), organic cation transporter 2 (OCT2), phosphodiesterase (PDE), peroxisome proliferator-activated receptor gamma (PPARγ), sphingosine 1-phosphate (S1P), store-operated calcium entry (SOCE), superoxide dismutase (SOD), potassium channel subfamily K member 2 (TREK1), transient receptor potential channel melastatin 8 (TRPM8).

IDrug	Approved diseases	Effects (Prevention^(a)^ or Treatment^(b)^)	Proposed mechanism	References
Acetazolamide	Edema	Oxaliplatin (acute)^(a)^	Inhibition of carbonic anhydrase	Potenzieri et al. (2020)
Alogliptin	Type 2 diabetes mellitus	Oxaliplatin (chronic)^(a)^, but Not oxaliplatin (acute), paclitaxel, and bortezomib	Unknown	Shigematsu et al. (2020)
Cimetidine	Heartburn, peptic ulcers	Oxaliplatin (acute)^(a)^	Inhibition of OCT2	Sprowl et al. (2013)
Dasatinib	Chronic myeloid leukemia Acute lymphoblastic leukemia	Oxaliplatin (acute)^(a)^	Inhibition of OCT2	Sprowl et al. (2016); Huang et al. (2020)
Diltiazem	Angina	Oxaliplatin (acute)^(a)^	Inhibition of calcium channel	Kawashiri et al. (2012)
Dimethyl fumarate	Multiple sclerosis	Oxaliplatin (chronic)^(a)^, but Not oxaliplatin (acute)	Activation of Nrf2 pathway	Miyagi et al. (2019)
Donepezil	Alzheimer’s disease	Oxaliplatin (chronic)^(a, b)^, and impairment of social interaction	Attenuation of reduction of SOD activity, and Activation of acetylcholine signaling	Ferrier et al. (2015); Kawashiri et al. (2019)
Ethosuximide	Absence epilepsy	Paclitaxel^(b)^	Inhibition of calcium channel	Flatters and Bennett (2004)
Exenatide	Type 2 diabetes mellitus	Facilitation of recovery from oxaliplatin (chronic)	Unknown	Fujita et al. (2015)
Fingolimod	Multiple sclerosis	Paclitaxel^(a, b)^	Modulation of S1P receptor	Janes et al. (2014)
Fluvestrant	Breast cancer	Oxaliplatin (chronic)^(a)^, but Not oxaliplatin (acute)	Unknown	Yamamoto et al. (2019)
Ifenprodil	Dizzy after cerebral hemorrhage	Oxaliplatin (chronic)^(b)^	Antagonism of NMDAR	Mihara et al. (2011)
Budilast	Asthma cerebrovascular disorders	Oxaliplatin (chronic)^(a, b)^, and impairment of memory paclitaxel-induced motor dysfunction	Inhibition of PDE inhibition of calpain protease activity	Mo et al. (2012); Johnston et al. (2017)
Memantine	Alzheimer’s disease	Oxaliplatin (chronic)^(b)^	Antagonism of NMDAR	Mihara et al. (2011)
Mexiletine	Arrhythmia	Oxaliplatin (acute, chronic)^(a, b)^	Inhibition of sodium channel	Egashira et al. (2010); Kawashiri et al. (2012)
Minoxidil	Hypertension	Paclitaxel^(a)^, and improvement of hair quality	Prevention of SOCE dysregulation	Chen et al. (2017)
Nifedipine	Hypertension	Oxaliplatin (acute)^(a)^	Inhibition of calcium channel	Kawashiri et al. (2012)
Nilotinib	Chronic myeloid leukemia	Paclitaxel^(a)^	Inhibition of OATP1B2	Leblanc et al. (2018)
Pirenzepine	Peptic ulcer	Paclitaxel^(a)^	Antagonism of muscarinic M1R	Calcutt et al. (2017)
Polaprezinc	Peptic ulcer	Paclitaxel^(a)^	Unknown	Tsutsumi et al. (2016)
Riluzole	Amyotrophic lateral sclerosis	Oxaliplatin (acute, chronic)^(a, b)^	Maintenance of glutamate homeostasis Activation of TREK-1 channel inhibition of TRPM8 activation	Yamamoto et al. (2017); Poupon et al. (2018); Yamamoto et al. (2018)
Rosiglitazone	Type 2 diabetes mellitus	Oxaliplatin (acute, chronic)^(a)^	Agonism of PPARγ	Zanardelli et al. (2014)
Tadalafil	Benign prostatic hyperplasia Erectile dysfunction	Oxaliplatin (acute, chronic)^(a, b)^	Inhibition of PDE5	Ogihara et al. (2019)
Telmisartan	Hypertension	Paclitaxel^(a, b)^	Inhibition of CYP2J	Sisignano et al. (2016a)
Thrombomodulin α	Disseminated intravascular coagulation	Oxaliplatin (chronic) and Paclitaxel^(a, b)^, but Not oxaliplatin (acute)	Degradation of HMGB1	Nishida et al. (2016); Tsubota et al. (2019)
Topiramate	Seizures	Oxaliplatin (acute)^(a)^	Inhibition of carbonic anhydrase	Potenzieri et al. (2020)

### Mechanism-Based Strategy

#### Neuronal Damage

CIPN induced by specific chemotherapeutic drugs is associated with neuronal damage related to sciatic nerve axonal degeneration in experimental rodent models. Several approved drugs for neurodegenerative diseases were investigated to determine whether they are useful for the prevention of chemotherapy-induced axonal degeneration and neuropathic pain. Dimethyl fumarate, a drug for multiple sclerosis, has anti-oxidative effects mediated via the Nrf2 pathway and exerts neuroprotective effects. Furthermore, dimethyl fumarate co-treatment ameliorated oxaliplatin-induced CIPN without affecting its anti-tumor activity (Miyagi et al., 2019). The acetylcholinesterase inhibitor donepezil, used for Alzheimer’s disease, was effective at preventing neuronal degeneration in *in vitro* and *in vivo* models of oxaliplatin-induced CIPN (Kawashiri et al., 2019). Acetylcholine signaling is involved in oxaliplatin- and paclitaxel-induced disorders of the nervous system (Di Cesare Mannelli et al., 2013b; Di Cesare Mannelli et al., 2014; Ferrier et al., 2015; Romero et al., 2017; Kyte et al., 2018; Toma et al., 2019). However, there are many acetylcholine-related receptor subtypes, and thus subtype-specific antagonists and agonists are needed to control acetylcholine signals for the treatment of CIPN.

#### Transporters of Chemotherapeutic Drugs

The inhibition of chemotherapeutic drug uptake into the nervous system is a promising strategy to prevent CIPN, although it is important to consider the potential effects on the anti-tumor activity. OCT2 or OATP1B2 are dominant transporters expressed in the DRG, which uptake oxaliplatin or paclitaxel, respectively. Cimetidine, a competitive inhibitor of OCT2, is approved for peptic ulcers and prevents acute cold allodynia after oxaliplatin injection (Sprowl et al., 2013). It was also reported that the tyrosine kinase inhibitor (TKI) nilotinib blocked OATP1B2 transport activity and suppressed the development of paclitaxel-induced neuropathic pain (Leblanc et al., 2018). Importantly, both OCT2 and OATP1B transporters are expressed at undetectable levels in tumor cell lines and tumor specimens, as confirmed by real-time PCR or RNA sequencing analysis (Sprowl et al., 2013; Leblanc et al., 2018).

#### Ion Channels

Based on the mechanism of TRPM8 upregulation, several ion channel inhibitors were examined for the prevention of oxaliplatin-induced cold allodynia. The co-administration of the L-type Ca^2+^ channel blockers, nifedipine and diltiazem, but not the T-type calcium channel inhibitor ethosuximide, prevented cold allodynia caused by oxaliplatin (or oxalate). In addition, the Na^+^ channel inhibitor mexiletine also prevented cold allodynia. These preventive effects were related to the inhibition of the Ca^2+^/NFAT/TRPM8 pathway (Kawashiri et al., 2012). A retrospective study of 69 patients receiving oxaliplatin-chemotherapy demonstrated that 26 patients co-treated with calcium channel blockers including nifedipine, amlodipine, and diltiazem, had a lower incidence of oxaliplatin-induced cold hypersensitivity compared with 43 control patients (*p* = 0.0438) (Tatsushima et al., 2013).

Topiramate and acetazolamide are known inhibitors of carbonic anhydrase, which facilitates intracellular pH homeostasis. These approved drugs inhibit cytosolic pH lowering and sensitization of TRPA1 by oxaliplatin, potentially prevent oxaliplatin-induced cold allodynia (Potenzieri et al., 2020).

#### Glutamate Signaling

Riluzole is a potential candidate for the prevention of oxaliplatin-induced CIPN via multiple mechanisms. Riluzole modulates the activities of several ion channels including TRPM8 and TREK1 and thus suppresses cold allodynia (Poupon et al., 2018; Yamamoto et al., 2018). In addition, oxaliplatin failed to disrupt glutamate homeostasis in the spinal cord after co-treatment with riluzole, which prevented oxaliplatin-induced mechanical allodynia (Yamamoto et al., 2017). Importantly, riluzole prevented oxaliplatin-induced morphological abnormalities of DRG neurons. In a recent study, riluzole co-administration was not effective against oxaliplatin-induced neuropathy (Trinh et al., 2020). However, this clinical trial had a limited sample size (placebo: 23 patients, riluzole: 25 patients), which means that further large-scale clinical studies are needed to clarify the effect of riluzole. The RILUZOX-01 study, a randomized, controlled, double-blind phase II clinical trial to evaluate the efficacy of riluzole on oxaliplatin-induced CIPN, is ongoing at present (Kerckhove et al., 2019).

Including riluzole, the treatment focused on the disruption of glutamate homeostasis and excessive signaling pathways in the CNS as potential strategies to treat CIPN. Several approved drugs such as memantine and ifenprodil have demonstrated efficacy in oxaliplatin-induced CIPN, especially mechanical allodynia, via the inhibition of NMDAR activity (Mihara et al., 2011; Shirahama et al., 2012).

#### Others

Cumulative evidence has indicated the pleiotropic effects of anti-diabetic drugs, especially glucagon-like peptide-1 (GLP-1)-related drugs such as GLP-1 receptor agonists and dipeptidyl peptidase 4 inhibitors. Several studies examined whether exenatide and alogliptin had beneficial effects against chemotherapy-induced neurodegeneration and neuropathic pain and demonstrated that these approved drugs attenuated axonal degeneration and mechanical allodynia after oxaliplatin administration in rats (Fujita et al., 2015; Shigematsu et al., 2020).

### Screening-Based Strategy

Over the last 5–10 years, approved drug-containing chemical libraries have been widely used to explore unknown pharmacological functions. One of the hallmarks of CIPN is axonal degeneration, which is reproduced in *in vitro* cellular models in the form of shortened neurite length or reduced neurite branches. Using *in vitro* models, several drug repositioning studies have discovered unexpected the neuroprotective effects of approved drugs.

#### Neuroprotective Drug

Fulvestrant, for the treatment of breast cancer, was screened from several small-molecule chemical libraries including more than 3,000 compounds and reduced oxaliplatin-induced neuronal damage in a cellular model (Yamamoto et al., 2019). Consistent with these *in vitro* effects, fulvestrant also exerted neuroprotective effects in an oxaliplatin-CIPN rodent model, by preventing sciatic nerve axonal degeneration and the development of mechanical allodynia (Yamamoto et al., 2019). However, the mechanism of this neuroprotective effect remains unclear. In a similar approach, minoxidil and pirenzepine, approved drugs for hypertension and alopecia, and peptic ulcers, respectively, were shown to be neuroprotective. Minoxidil has neuroprotective effects against paclitaxel in primary cultured DRG neurons and alleviated paclitaxel-induced CIPN by inhibiting neuroinflammation and remodeling the dysregulation of intracellular calcium homeostasis in DRG neurons (Chen et al., 2017). Minoxidil also improved hair quality after paclitaxel administration. Pirenzepine protected mice from peripheral neurodegeneration caused by chemotherapy, diabetes, and HIV envelope protein gp120 by antagonizing the muscarinic acetylcholine type 1 receptor, leading to improved mitochondrial dysfunction (Calcutt et al., 2017).

#### Inhibitor of OCT2 Transporter Activity

The screening of a large-scale bioactive compound library (8,086 compounds) identified the FDA-approved TKI dasatinib as a potent inhibitor of the OCT2 transporter, which is involved in oxaliplatin uptake to the DRG (Sprowl et al., 2016). OCT2 requires Yes1-mediating tyrosine phosphorylation; therefore, dasatinib treatment is sufficient to inhibit platinum accumulation in the DRG, which mitigates oxaliplatin-induced neuropathic pain.

#### Inhibitor of CYP2J Enzymatic Activity

Sisignano et al. reported paclitaxel treatment increased CYP2J expression in the DRG, which increased the levels of pronociceptive oxidized lipid mediators. Drug repositioning screening identified telmisartan, a widely-used angiotensin II receptor antagonist for hypertension, as a potent inhibitor of CYP2J, which prevents and reverses paclitaxel-induced neuropathic pain. In contrast, olmesartan, which belongs to the same pharmacological group, had no effect on CYP2J activity (Sisignano et al., 2016a). In addition, telmisartan is established as a selective antagonist of angiotensin II type 1 receptor, despite evidence that the similar drug candesartan exerts neuroprotective effects against vincristine-induced neuropathy via the angiotensin II type 2 receptor (Bessaguet et al., 2018). Thus, the analgesic effect of telmisartan may not be derived from its action on the angiotensin receptor.

### Future Expectations

#### Lipid-Related Molecules

Numerous mechanisms involved in CIPN have been revealed in recent years, and these might become potential therapeutic targets over the next decade. Biological lipids and related molecules are one of the most intriguing targets of drug discovery for CIPN prevention and treatment. In addition to telmisartan, fingolimod, an approved drug for the treatment of multiple sclerosis that modulates the S1P receptor, was also shown to be effective against paclitaxel-induced neuropathic pain (Janes et al., 2014). Furthermore, stimulation of cannabinoid type 2 receptor (CB2) exerted an analgesic effect against CIPN (Naguib et al., 2008; Rahn et al., 2008; Deng et al., 2015). The endocannabinoid 2-arachidonylglycerol, a ligand for CB2, is biosynthesized from membrane phospholipids and degraded by monoacylglycerol lipase. Similar to CB2 agonists, monoacylglycerol lipase inhibitors have a potential analgesic effect on chemotherapy-induced neuropathic pain (Brindisi et al., 2016; Curry et al., 2018). Therefore, based on these well-studied molecules, mechanism-based repositioning screening might identify and reposition many approved drugs as novel analgesics or novel preventative drugs for CIPN.

#### Fatty Acid-Derived Mediators

Omega-3 fatty acid and its related mediators also have antinociceptive properties. Although the effects of most of these mediators have been examined using nerve injury-induced neuropathic pain or inflammatory pain models, but not CIPN, they were shown to be effective against refractory pain syndromes (Xu et al., 2010; Ji et al., 2011; Xu et al., 2013). In particular, Serhan and colleagues identified new families of potent anti-inflammatory lipid mediators derived from omega-3 fatty acid, called “specialized pro-resolving mediators” (SPMs), which include resolvins, neuroprotectin, and maresins (Serhan et al., 2002; Serhan et al., 2008; Chen et al., 2018). SPMs have a wide range of analgesic effects against a variety of types of chronic pain. Among the docosahexaenoic acid metabolite series, the intrathecal injection of resolvin D_1_ (RvD_1_), RvD_2_, and RvD_5_ reduced paclitaxel-induced mechanical allodynia, which might be related to their inhibition of TRPV1 and/or TRPA1 (Luo et al., 2019b). Therefore, biological lipid mediators (especially SPMs) or lipid-related molecules (such as receptors and enzymes) are potential targets for the treatment of CIPN. However, not all omega-3 related mediators are anti-inflammatory. For example, an eicosapentaenoic acid-derived mediator 17,18-EEQ, sensitized TRPV1 and TRPA1 in a prostanoid receptor IP-dependent manner (Schafer et al., 2020).

#### Stem Cells

The utilization of stem cell-derived neurons has increased (Wainger et al., 2015; Schwartzentruber et al., 2018; Mis et al., 2019). Dolan et al. established a chemotherapy-induced neurotoxicity *in vitro* model using induced pluripotent stem cells differentiated into neurons (Wheeler et al., 2015; Wing et al., 2017). Although hydroxyurea and 5-fluorouracil, not known to cause CIPN, did not affect neurite morphology in this model, platinum agents, taxanes, and bortezomib reduced neurite outgrowth and processes, suggesting induced pluripotent stem cell-derived neurons might be useful for the screening of potential neuroprotective drug candidates. Thus, if patient-derived sensory neurons become easily available and are actively utilized in clinical practice, tailored therapy individualized for each patient could be realized, by screening and searching for drugs effective against CIPN. Furthermore, the intravenous injection of rat adipose-derived stem cells attenuated oxaliplatin-induced pain hypersensitivity in a rat model, and this analgesic effect lasted for 5 days (Di Cesare Mannelli et al., 2018). Thus, the utilization of stem cells including the injection of adipose-derived stem cells might be a novel therapeutic strategy. In addition, the elucidation of mechanisms related to this analgesic effect might enhance the analgesic efficacy of approved drugs.

#### Consideration for Oncological Safety

The drug repositioning studies reviewed here indicate that several approved drugs may have beneficial effects against CIPN in rodent models. However, careful attention to oncological safety with respect to interactions with both cancer cells and anticancer drugs is required when drugs are co-administered with neurotoxic anticancer drugs. Although most of the potential therapies reviewed here, particularly those for preventive use, have shown no effect on the anticancer activity of chemotherapeutic drugs in cancer cell lines or in tumor bearing animals, newly developed preventive and/or treatment strategy should be considered for translation into clinical practice.

## Conclusions

The prevention and treatment of existing CIPN remain unmet clinical needs. Recent studies have shown the involvement of a number of potential target molecules such as chemotherapeutic agent-uptake transporters (OCT2 and OATP1B2) and CYP2J, in the development of CIPN. Furthermore, drug repositioning studies have demonstrated the efficacy of several approved drugs such as TKIs, GLP-1-related agents, riluzole, and others, in rodent models of CIPN.

Drug repositioning studies are useful for demonstrating the action of a drug against CIPN, investigating the mechanisms of approved drugs, and discovering unexpected actions of approved drugs by the screening of chemical libraries. In addition, the screening of chemical libraries might help elucidate the mechanisms of CIPN, especially when investigating neuroprotective drugs. The known actions of hit compounds in neurodegenerative phenotypic assays might indicate how neurodegeneration occurs after chemotherapy.

Recent studies have reported potential therapeutic targets for CIPN including endogenous lipid-related molecules (such as SPMs), and the use of stem cells. In addition to the treatment of existing neuropathies, future trials should test drugs for the prevention of CIPN. The preclinical evidence reviewed here should be assessed in clinical trials, which we hope will result in the improved quality of life of cancer patients.

## Author Contributions

All authors listed have made a substantial, direct, and intellectual contribution to the work and approved it for publication.

## Funding

This work was supported by JSPS KAKENHI Grant Numbers JP19K16938 (SY) and JP17K08953 (NE).

## Conflict of Interest

The authors declare that the research was conducted in the absence of any commercial or financial relationships that could be construed as a potential conflict of interest.

## Abbreviations

ASCO, American Society of Clinical Oncology; CB2, cannabinoid type 2 receptor; CIPN, chemotherapy-induced peripheral neuropathy; CNS: central nervous system; CYP2J, cytochrome-P_450_-epoxygenase 2J; DRG, dorsal root ganglia; GLP-1, glucagon-like peptide-1; IL, interleukin; LPA, lysophosphatidic acid; NFAT, nuclear factor of activated T-cell; NMDAR, *N*-methyl-d-aspartate receptor; Nrf2, nuclear factor-erythroid-2-related factor 2; OATP1B2, organic anion-transporting polypeptide B2; OCT2, organic cation transporter 2; RvD, resolvin D; SPMs, specialized pro-resolving mediators; S1P, sphingosine 1-phosphate; TKI, tyrosine kinase inhibitor; TRPA, transient receptor potential ankyrin; TRPM, transient receptor potential melastatin; TRPV, transient receptor potential vanilloid.

## References

[B1] Alessandri-Haber N. Dina O. A. Yeh J. J. Parada C. A. Reichling D. B. Levine J. D. (2004). Transient receptor potential vanilloid 4 is essential in chemotherapy-induced neuropathic pain in the rat. J. Neurosci. 24 (18), 4444–4452. 10.1523/JNEUROSCI.0242-04.2004 15128858 PMC6729449

[B2] Bessaguet F. Danigo A. Bouchenaki H. Duchesne M. Magy L. Richard L. (2018). Neuroprotective effect of angiotensin II type 2 receptor stimulation in vincristine-induced mechanical allodynia. Pain 159 (12), 2538–2546. 10.1097/j.pain.0000000000001361 30086116

[B3] Bobylev I. Joshi A. R. Barham M. Ritter C. Neiss W. F. Höke A. (2015). Paclitaxel inhibits mRNA transport in axons. Neurobiol. Dis. 82, 321–331. 10.1016/j.nbd.2015.07.006 26188177

[B4] Boehmerle W. Huehnchen P. Lee S. L. L. Harms C. Endres M. (2018). TRPV4 inhibition prevents paclitaxel-induced neurotoxicity in preclinical models. Exp. Neurol. 306, 64–75. 10.1016/j.expneurol.2018.04.014 29715474

[B5] Boehmerle W. Zhang K. Sivula M. Heidrich F. M. Lee Y. Jordt S. E. (2007). Chronic exposure to paclitaxel diminishes phosphoinositide signaling by calpain-mediated neuronal calcium sensor-1 degradation. Proc. Natl. Acad. Sci. U.S.A. 104 (26), 11103–11108. 10.1073/pnas.0701546104 17581879 PMC1904151

[B6] Brewer J. R. Morrison G. Dolan M. E. Fleming G. F. (2016). Chemotherapy-induced peripheral neuropathy: current status and progress. Gynecol. Oncol. 140 (1), 176–183. 10.1016/j.ygyno.2015.11.011 26556766 PMC4698212

[B7] Brindisi M. Maramai S. Gemma S. Brogi S. Grillo A. Di Cesare Mannelli L. (2016). Development and pharmacological characterization of selective blockers of 2-arachidonoyl glycerol degradation with efficacy in rodent models of multiple sclerosis and pain. J. Med. Chem. 59 (6), 2612–2632. 10.1021/acs.jmedchem.5b01812 26888301

[B8] Calcutt N. A. Smith D. R. Frizzi K. Sabbir M. G. Chowdhury S. K. Mixcoatl-Zecuatl T. (2017). Selective antagonism of muscarinic receptors is neuroprotective in peripheral neuropathy. J. Clin. Invest. 127 (2), 608–622. 10.1172/JCI88321 28094765 PMC5272197

[B9] Cavaletti G. Marmiroli P. (2010). Chemotherapy-induced peripheral neurotoxicity. Nat. Rev. Neurol. 6 (12), 657–666. 10.1038/nrneurol.2010.160 21060341

[B10] Cavaletti G. Marmiroli P. (2020). Management of oxaliplatin-induced peripheral sensory neuropathy. Cancers 12 (6). 10.3390/cancers12061370 PMC7352541 32471028

[B11] Cavaletti G. Tredici G. Braga M. Tazzari S. (1995). Experimental peripheral neuropathy induced in adult rats by repeated intraperitoneal administration of taxol. Exp. Neurol. 133 (1), 64–72. 10.1006/exnr.1995.1008 7601264

[B12] Cavaletti G. Tredici G. Petruccioli M. G. Dondè E. Tredici P. Marmiroli P. (2001). Effects of different schedules of oxaliplatin treatment on the peripheral nervous system of the rat. Eur. J. Canc. 37 (18), 2457–2463. 10.1016/s0959-8049(01)00300-8 11720843

[B13] Chelini A. Brogi S. Paolino M. Di Capua A. Cappelli A. Giorgi G. (2017). Synthesis and biological evaluation of novel neuroprotective pyridazine derivatives as excitatory amino acid transporter 2 (EAAT2) activators. J. Med. Chem. 60 (12), 5216–5221. 10.1021/acs.jmedchem.7b00383 28525717

[B14] Chen G. Zhang Y. Q. Qadri Y. J. Serhan C. N. Ji R. R. (2018). Microglia in pain: detrimental and protective roles in pathogenesis and resolution of pain. Neuron 100 (6), 1292–1311. 10.1016/j.neuron.2018.11.009 30571942 PMC6312407

[B15] Chen Y. Yang C. Wang Z. J. (2011). Proteinase-activated receptor 2 sensitizes transient receptor potential vanilloid 1, transient receptor potential vanilloid 4, and transient receptor potential ankyrin 1 in paclitaxel-induced neuropathic pain. Neuroscience 193, 440–451. 10.1016/j.neuroscience.2011.06.085 21763756

[B16] Chen Y. F. Chen L. H. Yeh Y. M. Wu P. Y. Chen Y. F. Chang L. Y. (2017). Minoxidil is a potential neuroprotective drug for paclitaxel-induced peripheral neuropathy. Sci. Rep. 7, 45366 10.1038/srep45366 28349969 PMC5368986

[B17] Colloca L. Ludman T. Bouhassira D. Baron R. Dickenson A. H. Yarnitsky D. (2017). Neuropathic pain. Nat Rev Dis Primers 3, 17002 10.1038/nrdp.2017.2 28205574 PMC5371025

[B18] Costa R. Bicca M. A. Manjavachi M. N. Segat G. C. Dias F. C. Fernandes E. S. (2018). Kinin receptors sensitize TRPV4 channel and induce mechanical hyperalgesia: relevance to paclitaxel-induced peripheral neuropathy in mice. Mol. Neurobiol. 55 (3), 2150–2161. 10.1007/s12035-017-0475-9 28283888

[B19] Curry Z. A. Wilkerson J. L. Bagdas D. Kyte S. L. Patel N. Donvito G. (2018). Monoacylglycerol lipase inhibitors reverse paclitaxel-induced nociceptive behavior and proinflammatory markers in a mouse model of chemotherapy-induced neuropathy. J. Pharmacol. Exp. Therapeut. 366 (1), 169–183. 10.1124/jpet.117.245704 PMC6038031 29540562

[B20] Deng L. Cornett B. L. Mackie K. Hohmann A. G. (2015). CB1 knockout mice unveil sustained CB2-mediated antiallodynic effects of the mixed CB1/CB2 agonist CP55,940 in a mouse model of paclitaxel-induced neuropathic pain. Mol. Pharmacol. 88 (1), 64–74. 10.1124/mol.115.098483 25904556 PMC4468646

[B21] Descoeur J. Pereira V. Pizzoccaro A. Francois A. Ling B. Maffre V. (2011). Oxaliplatin-induced cold hypersensitivity is due to remodelling of ion channel expression in nociceptors. EMBO Mol. Med. 3 (5), 266–278. 10.1002/emmm.201100134 21438154 PMC3377073

[B22] Di Cesare Mannelli L. Pacini A. Bonaccini L. Zanardelli M. Mello T. Ghelardini C. (2013a). Morphologic features and glial activation in rat oxaliplatin-dependent neuropathic pain. J. Pain 14 (12), 1585–1600. 10.1016/j.jpain.2013.08.002 24135431

[B23] Di Cesare Mannelli L. Pacini A. Matera C. Zanardelli M. Mello T. De Amici M. (2014). Involvement of α7 nAChR subtype in rat oxaliplatin-induced neuropathy: effects of selective activation. Neuropharmacology 79, 37–48. 10.1016/j.neuropharm.2013.10.034 24225197

[B24] Di Cesare Mannelli L. Tenci B. Micheli L. Vona A. Corti F. Zanardelli M. (2018). Adipose-derived stem cells decrease pain in a rat model of oxaliplatin-induced neuropathy: role of VEGF-A modulation. Neuropharmacology 131, 166–175. 10.1016/j.neuropharm.2017.12.020 29241656

[B25] Di Cesare Mannelli L. Zanardelli M. Ghelardini C. (2013b). Nicotine is a pain reliever in trauma- and chemotherapy-induced neuropathy models. Eur. J. Pharmacol. 711 (1–3), 87–94. 10.1016/j.ejphar.2013.04.022 23648560

[B26] Durand J. P. Deplanque G. Montheil V. Gornet J. M. Scotte F. Mir O. (2012). Efficacy of venlafaxine for the prevention and relief of oxaliplatin-induced acute neurotoxicity: results of EFFOX, a randomized, double-blind, placebo-controlled phase III trial. Ann. Oncol. 23 (1), 200–205. 10.1093/annonc/mdr045 21427067

[B27] Egashira N. Hirakawa S. Kawashiri T. Yano T. Ikesue H. Oishi R. (2010). Mexiletine reverses oxaliplatin-induced neuropathic pain in rats. J. Pharmacol. Sci. 112 (4), 473–476. 10.1254/jphs.10012sc 20308797

[B28] El-Fatatry B. M. Ibrahim O. M. Hussien F. Z. Mostafa T. M. (2018). Role of metformin in oxaliplatin-induced peripheral neuropathy in patients with stage III colorectal cancer: randomized, controlled study. Int. J. Colorectal Dis. 33 (12), 1675–1683. 10.1007/s00384-018-3104-9 29931409

[B29] Ferrier J. Bayet-Robert M. Dalmann R. El Guerrab A. Aissouni Y. Graveron-Demilly D. (2015). Cholinergic neurotransmission in the posterior insular cortex is altered in preclinical models of neuropathic pain: key role of muscarinic M2 receptors in donepezil-induced antinociception. J. Neurosci. 35 (50), 16418–16430. 10.1523/JNEUROSCI.1537-15.2015 26674867 PMC4679823

[B30] Flatters S. J. Bennett G. J. (2004). Ethosuximide reverses paclitaxel- and vincristine-induced painful peripheral neuropathy. Pain 109 (1-2), 150–161. 10.1016/j.pain.2004.01.029 15082137

[B31] Fujita S. Hirota T. Sakiyama R. Baba M. Ieiri I. (2019). Identification of drug transporters contributing to oxaliplatin-induced peripheral neuropathy. J. Neurochem. 148 (3), 373–385. 10.1111/jnc.14607 30295925

[B32] Fujita S. Ushio S. Ozawa N. Masuguchi K. Kawashiri T. Oishi R. (2015). Exenatide facilitates recovery from oxaliplatin-induced peripheral neuropathy in rats. PLoS One 10 (11), e0141921 10.1371/journal.pone.0141921 26536615 PMC4633148

[B33] Gauchan P. Andoh T. Kato A. Kuraishi Y. (2009). Involvement of increased expression of transient receptor potential metastatic 8 in oxaliplatin-induced cold allodynia in mice. Neurosci. Lett. 458 (2), 93–95. 10.1016/j.neulet.2009.04.029 19375484

[B34] Grolleau F. Gamelin L. Boisdron-Celle M. Lapied B. Pelhate M. Gamelin E. (2001). A possible explanation for a neurotoxic effect of the anticancer agent oxaliplatin on neuronal voltage-gated sodium channels. J. Neurophysiol. 85 (5), 2293–2297. 10.1152/jn.2001.85.5.2293 11353042

[B35] Hara T. Chiba T. Abe K. Makabe A. Ikeno S. Kawakami K. (2013). Effect of paclitaxel on transient receptor potential vanilloid 1 in rat dorsal root ganglion. Pain 154 (6), 882–889. 10.1016/j.pain.2013.02.023 23602343

[B36] Hershman D. L. Lacchetti C. Dworkin R. H. Lavoie Smith E. M. Bleeker J. Cavaletti G. (2014). Prevention and management of chemotherapy-induced peripheral neuropathy in survivors of adult cancers: American Society of Clinical Oncology clinical practice guideline. J. Clin. Oncol. 32 (18), 1941–1967. 10.1200/JCO.2013.54.0914 24733808

[B37] Holmes J. Stanko J. Varchenko M. Ding H. Madden V. J. Bagnell C. R. (1998). Comparative neurotoxicity of oxaliplatin, cisplatin, and ormaplatin in a Wistar rat model. Toxicol. Sci. 46 (2), 342–351. 10.1006/toxs.1998.2558 10048138

[B38] Huang K. M. Leblanc A. F. Uddin M. E. Kim J. Y. Chen M. Eisenmann E. D. (2020). Neuronal uptake transporters contribute to oxaliplatin neurotoxicity in mice. J. Clin. Invest. 130 (9), 4601–4606. 10.1172/JCI136796 32484793 PMC7456253

[B39] Imai S. Koyanagi M. Azimi Z. Nakazato Y. Matsumoto M. Ogihara T. (2017). Taxanes and platinum derivatives impair Schwann cells via distinct mechanisms. Sci. Rep. 7 (1), 5947 10.1038/s41598-017-05784-1 28729624 PMC5519765

[B40] Ito N. Sakai A. Miyake N. Maruyama M. Iwasaki H. Miyake K. (2017). miR-15b mediates oxaliplatin-induced chronic neuropathic pain through BACE1 down-regulation. Br. J. Pharmacol. 174 (5), 386–395. 10.1111/bph.13698 28012171 PMC5301044

[B41] Janes K. Little J. W. Li C. Bryant L. Chen C. Chen Z. (2014). The development and maintenance of paclitaxel-induced neuropathic pain require activation of the sphingosine 1-phosphate receptor subtype 1. J. Biol. Chem. 289 (30), 21082–21097. 10.1074/jbc.M114.569574 24876379 PMC4110312

[B42] Ji R. R. Xu Z. Z. Strichartz G. Serhan C. N. (2011). Emerging roles of resolvins in the resolution of inflammation and pain. Trends Neurosci. 34 (11), 599–609. 10.1016/j.tins.2011.08.005 21963090 PMC3200462

[B43] Johnston I. N. Tan M. Cao J. Matsos A. Forrest D. R. L. Si E. (2017). Ibudilast reduces oxaliplatin-induced tactile allodynia and cognitive impairments in rats. Behav. Brain Res. 334, 109–118. 10.1016/j.bbr.2017.07.021 28739131

[B44] Jong N. N. Nakanishi T. Liu J. J. Tamai I. McKeage M. J. (2011). Oxaliplatin transport mediated by organic cation/carnitine transporters OCTN1 and OCTN2 in overexpressing human embryonic kidney 293 cells and rat dorsal root ganglion neurons. J. Pharmacol. Exp. Therapeut. 338 (2), 537–547. 10.1124/jpet.111.181297 21606177

[B45] Kawashiri T. Egashira N. Kurobe K. Tsutsumi K. Yamashita Y. Ushio S. (2012). L type Ca²+ channel blockers prevent oxaliplatin-induced cold hyperalgesia and TRPM8 overexpression in rats. Mol. Pain 8, 7 10.1186/1744-8069-8-7 22292988 PMC3285049

[B46] Kawashiri T. Shimizu S. Shigematsu N. Kobayashi D. Shimazoe T. (2019). Donepezil ameliorates oxaliplatin-induced peripheral neuropathy via a neuroprotective effect. J. Pharmacol. Sci. 140 (3), 291–294. 10.1016/j.jphs.2019.05.009 31377017

[B47] Kerckhove N. Busserolles J. Stanbury T. Pereira B. Plence V. Bonnetain F. (2019). Effectiveness assessment of riluzole in the prevention of oxaliplatin-induced peripheral neuropathy: RILUZOX-01: protocol of a randomised, parallel, controlled, double-blind and multicentre study by the UNICANCER-AFSOS Supportive Care intergroup. BMJ Open 9 (6), e027770 10.1136/bmjopen-2018-027770 PMC6561607 31182448

[B48] Krukowski K. Eijkelkamp N. Laumet G. Hack C. E. Li Y. Dougherty P. M. (2016). CD8+ T cells and endogenous IL-10 are required for resolution of chemotherapy-induced neuropathic pain. J. Neurosci. 36 (43), 11074–11083. 10.1523/JNEUROSCI.3708-15.2016 27798187 PMC5098842

[B49] Kyte S. L. Toma W. Bagdas D. Meade J. A. Schurman L. D. Lichtman A. H. (2018). Nicotine prevents and reverses paclitaxel-induced mechanical allodynia in a mouse model of CIPN. J. Pharmacol. Exp. Therapeut. 364 (1), 110–119. 10.1124/jpet.117.243972 PMC5738719 29042416

[B50] Leblanc A. F. Sprowl J. A. Alberti P. Chiorazzi A. Arnold W. D. Gibson A. A. (2018). OATP1B2 deficiency protects against paclitaxel-induced neurotoxicity. J. Clin. Invest. 128 (2), 816–825. 10.1172/JCI96160 29337310 PMC5785270

[B51] Ledeboer A. Jekich B. M. Sloane E. M. Mahoney J. H. Langer S. J. Milligan E. D. (2007). Intrathecal interleukin-10 gene therapy attenuates paclitaxel-induced mechanical allodynia and proinflammatory cytokine expression in dorsal root ganglia in rats. Brain Behav. Immun. 21 (5), 686–698. 10.1016/j.bbi.2006.10.012 17174526 PMC2063454

[B52] Li D. Huang Z. Z. Ling Y. Z. Wei J. Y. Cui Y. Zhang X. Z. (2015). Up-regulation of CX3CL1 via nuclear factor-κb-dependent histone acetylation is involved in paclitaxel-induced peripheral neuropathy. Anesthesiology 122 (5), 1142–1151. 10.1097/ALN.0000000000000560 25494456

[B53] Li Y. North R. Y. Rhines L. D. Tatsui C. E. Rao G. Edwards D. D. (2018). DRG voltage-gated sodium channel 1.7 is upregulated in paclitaxel-induced neuropathy in rats and in humans with neuropathic pain. J. Neurosci. 38 (5), 1124–1136. 10.1523/JNEUROSCI.0899-17.2017 29255002 PMC5792474

[B54] LoCoco P. M. Risinger A. L. Smith H. R. Chavera T. S. Berg K. A. Clarke W. P. (2017). Pharmacological augmentation of nicotinamide phosphoribosyltransferase (NAMPT) protects against paclitaxel-induced peripheral neuropathy. Elife 6, e29626 10.7554/eLife.29626 29125463 PMC5701795

[B55] Loprinzi C. L. Lacchetti C. Bleeker J. Cavaletti G. Chauhan C. Hertz D. L. (2020). Prevention and management of chemotherapy-induced peripheral neuropathy in survivors of adult cancers: ASCO guideline update. J. Clin. Oncol. 32 (18), 1961–1967. 10.1200/JCO.20.01399 32663120

[B56] Luo H. Liu H. Z. Zhang W. W. Matsuda M. Lv N. Chen G. (2019a). Interleukin-17 regulates neuron-glial communications, synaptic transmission, and neuropathic pain after chemotherapy. Cell Rep. 29 (8), 2384–e2385. 10.1016/j.celrep.2019.10.085 31747607

[B57] Luo X. Gu Y. Tao X. Serhan C. N. Ji R. R. (2019b). Resolvin D5 inhibits neuropathic and inflammatory pain in male but not female mice: distinct actions of D-series resolvins in chemotherapy-induced peripheral neuropathy. Front. Pharmacol. 10, 745 10.3389/fphar.2019.00745 31333464 PMC6624779

[B58] Manjavachi M. N. Passos G. F. Trevisan G. Araújo S. B. Pontes J. P. Fernandes E. S. (2019). Spinal blockage of CXCL1 and its receptor CXCR2 inhibits paclitaxel-induced peripheral neuropathy in mice. Neuropharmacology 151, 136–143. 10.1016/j.neuropharm.2019.04.014 30991054

[B59] Mantyh P. W. (2006). Cancer pain and its impact on diagnosis, survival and quality of life. Nat. Rev. Neurosci. 7 (10), 797–809. 10.1038/nrn1914 16988655

[B60] Mihara Y. Egashira N. Sada H. Kawashiri T. Ushio S. Yano T. (2011). Involvement of spinal NR2B-containing NMDA receptors in oxaliplatin-induced mechanical allodynia in rats. Mol. Pain 7, 8 10.1186/1744-8069-7-8 21247499 PMC3033350

[B61] Mis M. A. Yang Y. Tanaka B. S. Gomis-Perez C. Liu S. Dib-Hajj F. (2019). Resilience to pain: a peripheral component identified using induced pluripotent stem cells and dynamic clamp. J. Neurosci. 39 (3), 382–392. 10.1523/JNEUROSCI.2433-18.2018 30459225 PMC6335750

[B62] Miyagi A. Kawashiri T. Shimizu S. Shigematsu N. Kobayashi D. Shimazoe T. (2019). Dimethyl fumarate attenuates oxaliplatin-induced peripheral neuropathy without affecting the anti-tumor activity of oxaliplatin in rodents. Biol. Pharm. Bull. 42 (4), 638–644. 10.1248/bpb.b18-00855 30930422

[B63] Miyake T. Nakamura S. Zhao M. So K. Inoue K. Numata T. (2016). Cold sensitivity of TRPA1 is unveiled by the prolyl hydroxylation blockade-induced sensitization to ROS. Nat. Commun. 7, 12840 10.1038/ncomms12840 27628562 PMC5027619

[B64] Mo M. Erdelyi I. Szigeti-Buck K. Benbow J. H. Ehrlich B. E. (2012). Prevention of paclitaxel-induced peripheral neuropathy by lithium pretreatment. Faseb. J. 26 (11), 4696–4709. 10.1096/fj.12-214643 22889832 PMC3475250

[B65] Naguib M. Diaz P. Xu J. J. Astruc-Diaz F. Craig S. Vivas-Mejia P. (2008). MDA7: a novel selective agonist for CB2 receptors that prevents allodynia in rat neuropathic pain models. Br. J. Pharmacol. 155 (7), 1104–1116. 10.1038/bjp.2008.340 18846037 PMC2597252

[B66] Nassini R. Gees M. Harrison S. De Siena G. Materazzi S. Moretto N. (2011). Oxaliplatin elicits mechanical and cold allodynia in rodents via TRPA1 receptor stimulation. Pain 152 (7), 1621–1631. 10.1016/j.pain.2011.02.051 21481532

[B67] Nishida K. Takeuchi K. Hosoda A. Sugano S. Morisaki E. Ohishi A. (2018). Ergothioneine ameliorates oxaliplatin-induced peripheral neuropathy in rats. Life Sci. 207, 516–524. 10.1016/j.lfs.2018.07.006 29981320

[B68] Nishida T. Tsubota M. Kawaishi Y. Yamanishi H. Kamitani N. Sekiguchi F. (2016). Involvement of high mobility group box 1 in the development and maintenance of chemotherapy-induced peripheral neuropathy in rats. Toxicology 365, 48–58. 10.1016/j.tox.2016.07.016 27474498

[B69] Ochi-ishi R. Nagata K. Inoue T. Tozaki-Saitoh H. Tsuda M. Inoue K. (2014). Involvement of the chemokine CCL3 and the purinoceptor P2X7 in the spinal cord in paclitaxel-induced mechanical allodynia. Mol. Pain 10, 53 10.1186/1744-8069-10-53 25127716 PMC4141668

[B70] Ogihara T. Nakagawa T. Hayashi M. Koyanagi M. Yonezawa A. Omura T. (2019). Improvement of peripheral vascular impairment by a phosphodiesterase type 5 inhibitor tadalafil prevents oxaliplatin-induced peripheral neuropathy in mice. J. Pharmacol. Sci. 141 (4), 131–138. 10.1016/j.jphs.2019.10.005 31734027

[B71] Park S. B. Goldstein D. Krishnan A. V. Lin C. S. Friedlander M. L. Cassidy J. (2013). Chemotherapy-induced peripheral neurotoxicity: a critical analysis. CA Cancer J Clin 63 (6), 419–437. 10.3322/caac.21204 24590861

[B72] Pease-Raissi S. E. Pazyra-Murphy M. F. Li Y. Wachter F. Fukuda Y. Fenstermacher S. J. (2017). Paclitaxel reduces axonal Bclw to initiate IP3R1-dependent axon degeneration. Neuron 96 (2), 373–e376. 10.1016/j.neuron.2017.09.034 29024661 PMC5680044

[B73] Pereira V. Busserolles J. Christin M. Devilliers M. Poupon L. Legha W. (2014). Role of the TREK2 potassium channel in cold and warm thermosensation and in pain perception. Pain 155 (12), 2534–2544. 10.1016/j.pain.2014.09.013 25239074

[B74] Peters C. M. Jimenez-Andrade J. M. Jonas B. M. Sevcik M. A. Koewler N. J. Ghilardi J. R. (2007). Intravenous paclitaxel administration in the rat induces a peripheral sensory neuropathy characterized by macrophage infiltration and injury to sensory neurons and their supporting cells. Exp. Neurol. 203 (1), 42–54. 10.1016/j.expneurol.2006.07.022 17005179

[B75] Potenzieri A. Riva B. Rigolio R. Chiorazzi A. Pozzi E. Ballarini E. (2020). Oxaliplatin-induced neuropathy occurs through impairment of haemoglobin proton buffering and is reversed by carbonic anhydrase inhibitors. Pain 161 (2), 405–415. 10.1097/j.pain.0000000000001722 31634341

[B76] Poupon L. Lamoine S. Pereira V. Barriere D. A. Lolignier S. Giraudet F. (2018). Targeting the TREK-1 potassium channel via riluzole to eliminate the neuropathic and depressive-like effects of oxaliplatin. Neuropharmacology 140, 43–61. 10.1016/j.neuropharm.2018.07.026 30056126

[B77] Rahn E. J. Zvonok A. M. Thakur G. A. Khanolkar A. D. Makriyannis A. Hohmann A. G. (2008). Selective activation of cannabinoid CB2 receptors suppresses neuropathic nociception induced by treatment with the chemotherapeutic agent paclitaxel in rats. J. Pharmacol. Exp. Therapeut. 327 (2), 584–591. 10.1124/jpet.108.141994 PMC2682949 18664590

[B78] Resta F. Micheli L. Laurino A. Spinelli V. Mello T. Sartiani L. (2018). Selective HCN1 block as a strategy to control oxaliplatin-induced neuropathy. Neuropharmacology 131, 403–413. 10.1016/j.neuropharm.2018.01.014 29339292

[B79] Riva B. Dionisi M. Potenzieri A. Chiorazzi A. Cordero-Sanchez C. Rigolio R. (2018). Oxaliplatin induces pH acidification in dorsal root ganglia neurons. Sci. Rep. 8 (1), 15084 10.1038/s41598-018-33508-6 30305703 PMC6180129

[B80] Romero H. K. Christensen S. B. Di Cesare Mannelli L. Gajewiak J. Ramachandra R. Elmslie K. S. (2017). Inhibition of α9α10 nicotinic acetylcholine receptors prevents chemotherapy-induced neuropathic pain. Proc. Natl. Acad. Sci. U.S.A. 114 (10), E1825–E1832. 10.1073/pnas.1621433114 28223528 PMC5347537

[B81] Sakurai M. Egashira N. Kawashiri T. Yano T. Ikesue H. Oishi R. (2009). Oxaliplatin-induced neuropathy in the rat: involvement of oxalate in cold hyperalgesia but not mechanical allodynia. Pain 147 (1–3), 165–174. 10.1016/j.pain.2009.09.003 19782472

[B82] Schafer S. M. G. Sendetski M. Angioni C. Nüsing R. Geisslinger G. Scholich K. (2020). The omega-3 lipid 17,18-EEQ sensitizes TRPV1 and TRPA1 in sensory neurons through the prostacyclin receptor (IP). Neuropharmacology 166, 107952 10.1016/j.neuropharm.2020.107952 31955004

[B83] Schwartzentruber J. Foskolou S. Kilpinen H. Rodrigues J. Alasoo K. Knights A. J. (2018). Molecular and functional variation in iPSC-derived sensory neurons. Nat. Genet. 50 (1), 54–61. 10.1038/s41588-017-0005-8 29229984 PMC5742539

[B84] Serhan C. N. Chiang N. Van Dyke T. E. (2008). Resolving inflammation: dual anti-inflammatory and pro-resolution lipid mediators. Nat. Rev. Immunol. 8 (5), 349–361. 10.1038/nri2294 18437155 PMC2744593

[B85] Serhan C. N. Hong S. Gronert K. Colgan S. P. Devchand P. R. Mirick G. (2002). Resolvins: a family of bioactive products of omega-3 fatty acid transformation circuits initiated by aspirin treatment that counter proinflammation signals. J. Exp. Med. 196 (8), 1025–1037. 10.1084/jem.20020760 12391014 PMC2194036

[B86] Shigematsu N. Kawashiri T. Kobayashi D. Shimizu S. Mine K. Hiromoto S. (2020). Neuroprotective effect of alogliptin on oxaliplatin-induced peripheral neuropathy *in vivo* and *in vitro* . Sci. Rep. 10 (1), 6734 10.1038/s41598-020-62738-w 32317735 PMC7174301

[B87] Shirahama M. Ushio S. Egashira N. Yamamoto S. Sada H. Masuguchi K. (2012). Inhibition of Ca2+/calmodulin-dependent protein kinase II reverses oxaliplatin-induced mechanical allodynia in rats. Mol. Pain 8, 26 10.1186/1744-8069-8-26 22510452 PMC3384243

[B88] Sisignano M. Angioni C. Park C. K. Meyer Dos Santos S. Jordan H. Kuzikov M. (2016a). Targeting CYP2J to reduce paclitaxel-induced peripheral neuropathic pain. Proc. Natl. Acad. Sci. U.S.A. 113 (44), 12544–12549. 10.1073/pnas.1613246113 27791151 PMC5098623

[B89] Sisignano M. Baron R. Scholich K. Geisslinger G. (2014). Mechanism-based treatment for chemotherapy-induced peripheral neuropathic pain. Nat. Rev. Neurol. 10 (12), 694–707. 10.1038/nrneurol.2014.211 25366108

[B90] Sisignano M. Parnham M. J. Geisslinger G. (2016b). Drug repurposing for the development of novel analgesics. Trends Pharmacol. Sci. 37 (3), 172–183. 10.1016/j.tips.2015.11.006 26706620

[B91] Smith J. A. Slusher B. S. Wozniak K. M. Farah M. H. Smiyun G. Wilson L. (2016). Structural basis for induction of peripheral neuropathy by microtubule-targeting cancer drugs. Cancer Res 76 (17), 5115–5123. 10.1158/0008-5472.CAN-15-3116 27488522

[B92] So K. Haraguchi K. Asakura K. Isami K. Sakimoto S. Shirakawa H. (2015). Involvement of TRPM2 in a wide range of inflammatory and neuropathic pain mouse models. J. Pharmacol. Sci. 127 (3), 237–243. 10.1016/j.jphs.2014.10.003 25837919

[B93] Sprowl J. A. Ciarimboli G. Lancaster C. S. Giovinazzo H. Gibson A. A. Du G. (2013). Oxaliplatin-induced neurotoxicity is dependent on the organic cation transporter OCT2. Proc. Natl. Acad. Sci. U.S.A. 110 (27), 11199–11204. 10.1073/pnas.1305321110 23776246 PMC3704038

[B94] Sprowl J. A. Ong S. S. Gibson A. A. Hu S. Du G. Lin W. (2016). A phosphotyrosine switch regulates organic cation transporters. Nat. Commun. 7, 10880 10.1038/ncomms10880 26979622 PMC4799362

[B95] Tatsushima Y. Egashira N. Narishige Y. Fukui S. Kawashiri T. Yamauchi Y. (2013). Calcium channel blockers reduce oxaliplatin-induced acute neuropathy: a retrospective study of 69 male patients receiving modified FOLFOX6 therapy. Biomed. Pharmacother. 67 (1), 39–42. 10.1016/j.biopha.2012.10.006 23206755

[B96] Toma W. Kyte S. L. Bagdas D. Jackson A. Meade J. A. Rahman F. (2019). The α7 nicotinic receptor silent agonist R-47 prevents and reverses paclitaxel-induced peripheral neuropathy in mice without tolerance or altering nicotine reward and withdrawal. Exp. Neurol. 320, 113010 10.1016/j.expneurol.2019.113010 31299179 PMC6708482

[B97] Trevisan G. Materazzi S. Fusi C. Altomare A. Aldini G. Lodovici M. (2013). Novel therapeutic strategy to prevent chemotherapy-induced persistent sensory neuropathy by TRPA1 blockade. Cancer Res 73 (10), 3120–3131. 10.1158/0008-5472.CAN-12-4370 23477783

[B98] Trinh T. Park S. B. Murray J. Pickering H. Lin C. S. Martin A. (2020). Neu-horizons: neuroprotection and therapeutic use of riluzole for the prevention of oxaliplatin-induced neuropathy-a randomised controlled trial. Support. Care Cancer. 10.1007/s00520-020-05591-x 32607598

[B99] Tsubota M. Fukuda R. Hayashi Y. Miyazaki T. Ueda S. Yamashita R. (2019). Role of non-macrophage cell-derived HMGB1 in oxaliplatin-induced peripheral neuropathy and its prevention by the thrombin/thrombomodulin system in rodents: negative impact of anticoagulants. J. Neuroinflammation 16 (1), 199 10.1186/s12974-019-1581-6 31666085 PMC6822350

[B100] Tsutsumi K. Kaname T. Shiraishi H. Kawashiri T. Egashira N. (2016). Polaprezinc reduces paclitaxel-induced peripheral neuropathy in rats without affecting anti-tumor activity. J. Pharmacol. Sci. 131 (2), 146–149. 10.1016/j.jphs.2016.04.019 27262900

[B101] Tsutsumi K. Yamashita Y. Ushio S. Kawashiri T. Kaname T. Fujita S. (2014). Oxaliplatin induces hypomyelination and reduced neuregulin 1 expression in the rat sciatic nerve. Neurosci. Res. 80, 86–90. 10.1016/j.neures.2014.02.004 24530887

[B102] Uchida H. Nagai J. Ueda H. (2014). Lysophosphatidic acid and its receptors LPA1 and LPA3 mediate paclitaxel-induced neuropathic pain in mice. Mol. Pain 10, 71 10.1186/1744-8069-10-71 25411045 PMC4246549

[B103] Wainger B. J. Buttermore E. D. Oliveira J. T. Mellin C. Lee S. Saber W. A. (2015). Modeling pain *in vitro* using nociceptor neurons reprogrammed from fibroblasts. Nat. Neurosci. 18 (1), 17–24. 10.1038/nn.3886 25420066 PMC4429606

[B104] Wang M. S. Davis A. A. Culver D. G. Glass J. D. (2002). WldS mice are resistant to paclitaxel (taxol) neuropathy. Ann. Neurol. 52 (4), 442–447. 10.1002/ana.10300 12325073

[B105] Wang M. S. Davis A. A. Culver D. G. Wang Q. Powers J. C. Glass J. D. (2004). Calpain inhibition protects against Taxol-induced sensory neuropathy. Brain 127 (Pt 3), 671–679. 10.1093/brain/awh078 14761904

[B106] Warwick R. A. Hanani M. (2013). The contribution of satellite glial cells to chemotherapy-induced neuropathic pain. Eur. J. Pain 17 (4), 571–580. 10.1002/j.1532-2149.2012.00219.x 23065831

[B107] Wheeler H. E. Wing C. Delaney S. M. Komatsu M. Dolan M. E. (2015). Modeling chemotherapeutic neurotoxicity with human induced pluripotent stem cell-derived neuronal cells. PLoS One 10 (2), e0118020 10.1371/journal.pone.0118020 25689802 PMC4331516

[B108] Wing C. Komatsu M. Delaney S. M. Krause M. Wheeler H. E. Dolan M. E. (2017). Application of stem cell derived neuronal cells to evaluate neurotoxic chemotherapy. Stem Cell Res. 22, 79–88. 10.1016/j.scr.2017.06.006 28645005 PMC5737666

[B109] Wozniak K. M. Vornov J. J. Wu Y. Liu Y. Carozzi V. A. Rodriguez-Menendez V. (2018). Peripheral neuropathy induced by microtubule-targeted chemotherapies: insights into acute injury and long-term recovery. Cancer Res 78 (3), 817–829. 10.1158/0008-5472.CAN-17-1467 29191802 PMC5811354

[B110] Xiao W. H. Zheng H. Bennett G. J. (2012). Characterization of oxaliplatin-induced chronic painful peripheral neuropathy in the rat and comparison with the neuropathy induced by paclitaxel. Neuroscience 203, 194–206. 10.1016/j.neuroscience.2011.12.023 22200546 PMC3273641

[B111] Xu Z. Z. Berta T. Ji R. R. (2013). Resolvin E1 inhibits neuropathic pain and spinal cord microglial activation following peripheral nerve injury. J. Neuroimmune Pharmacol. 8 (1), 37–41. 10.1007/s11481-012-9394-8 22878925 PMC3671904

[B112] Xu Z. Z. Zhang L. Liu T. Park J. Y. Berta T. Yang R. (2010). Resolvins RvE1 and RvD1 attenuate inflammatory pain via central and peripheral actions. Nat. Med. 16 (5), 592–597. 10.1038/nm.2123 20383154 PMC2866054

[B113] Yamamoto S. Egashira N. Tsuda M. Masuda S. (2018). Riluzole prevents oxaliplatin-induced cold allodynia via inhibition of overexpression of transient receptor potential metastatic 8 in rats. J. Pharmacol. Sci. 138 (3), 214–217. 10.1016/j.jphs.2018.10.006 30409714

[B114] Yamamoto S. Ushio S. Egashira N. Kawashiri T. Mitsuyasu S. Higuchi H. (2017). Excessive spinal glutamate transmission is involved in oxaliplatin-induced mechanical allodynia: a possibility for riluzole as a prophylactic drug. Sci. Rep. 7 (1), 9661 10.1038/s41598-017-08891-1 28851920 PMC5574967

[B115] Yamamoto S. Yamashita T. Ito M. Caaveiro J. M. M. Egashira N. Tozaki-Saitoh H. (2019). New pharmacological effect of fulvestrant to prevent oxaliplatin-induced neurodegeneration and mechanical allodynia in rats. Int. J. Canc. 145 (8), 2107–2113. 10.1002/ijc.32043 30515800

[B116] Yang Y. Luo L. Cai X. Fang Y. Wang J. Chen G. (2018). Nrf2 inhibits oxaliplatin-induced peripheral neuropathy via protection of mitochondrial function. Free Radic. Biol. Med. 120, 13–24. 10.1016/j.freeradbiomed.2018.03.007 29530794

[B117] Yoon S. Y. Robinson C. R. Zhang H. Dougherty P. M. (2013). Spinal astrocyte gap junctions contribute to oxaliplatin-induced mechanical hypersensitivity. J. Pain 14 (2), 205–214. 10.1016/j.jpain.2012.11.002 23374942 PMC3564051

[B118] Zanardelli M. Micheli L. Cinci L. Failli P. Ghelardini C. Di Cesare Mannelli L. (2014). Oxaliplatin neurotoxicity involves peroxisome alterations. PPARγ agonism as preventive pharmacological approach. PLoS One 9 (7), e102758 10.1371/journal.pone.0102758 25036594 PMC4103888

[B119] Zhang H. Li Y. de Carvalho-Barbosa M. Kavelaars A. Heijnen C. J. Albrecht P. J. (2016). Dorsal root ganglion infiltration by macrophages contributes to paclitaxel chemotherapy-induced peripheral neuropathy. J. Pain 17 (7), 775–786. 10.1016/j.jpain.2016.02.011 26979998 PMC4939513

[B120] Zhang H. Yoon S. Y. Zhang H. Dougherty P. M. (2012). Evidence that spinal astrocytes but not microglia contribute to the pathogenesis of Paclitaxel-induced painful neuropathy. J. Pain 13 (3), 293–303. 10.1016/j.jpain.2011.12.002 22285612 PMC3294066

[B121] Zhao M. Isami K. Nakamura S. Shirakawa H. Nakagawa T. Kaneko S. (2012). Acute cold hypersensitivity characteristically induced by oxaliplatin is caused by the enhanced responsiveness of TRPA1 in mice. Mol. Pain 8, 55 10.1186/1744-8069-8-55 22839205 PMC3495669

[B122] Zheng H. Xiao W. H. Bennett G. J. (2011). Functional deficits in peripheral nerve mitochondria in rats with paclitaxel- and oxaliplatin-evoked painful peripheral neuropathy. Exp. Neurol. 232 (2), 154–161. 10.1016/j.expneurol.2011.08.016 21907196 PMC3202047

